# Egg shelf life can be extended using varied proportions of polyvinyl alcohol/chitosan composite coatings

**DOI:** 10.1002/fsn3.3394

**Published:** 2023-05-07

**Authors:** Dariush Khademi Shurmasti, Pezhman Riazi Kermani, Maryam Sarvarian, Chinaza Godswill Awuchi

**Affiliations:** ^1^ Department of Agriculture‐Food Science & Technology, Savadkooh Branch Islamic Azad University Savadkooh Iran; ^2^ School of Natural and Applied Sciences Kampala International University Kampala Uganda

**Keywords:** biodegradable, blended coating, egg internal quality, eggshell morphology

## Abstract

Using biopolymers in the form of edible coating as egg eco‐friendly packaging is a progressive approach. The blending of biopolymers is one of the procedures for overcoming mechanical weakness and benefiting from their maximum synergistic effect. Aiming to determine the relative ratios of chitosan (CH 4 w/v%) and polyvinyl alcohol (PVA 5 w/v%) in the composition of blended coatings, an experiment was conducted with six treatments (*r* = 3) including different ratios of CH/PVA (0:0; control, 100:0, 75:25, 50:50, 25:75, and 0:100) wt% in a period of 4 weeks of egg storage at ambient temperature, emphasizing eggshell barrier properties. Based on the eggshell analysis result, SEM images and FTIR spectra demonstrated that the components were firmly integrated into the blended coatings as well as had more intertwined than their pure ones, which was also reflected in the evaluation results of their internal quality parameters. In addition, the results showed that by enhancing the ratio of polyvinyl alcohol from 25 to 75 wt%, the blended coating barrier efficiency was relatively improved (*p* < .05). Meanwhile, the lowest percentage of weight loss (0.57 ± 0.08%), pH value of albumin (8.30 ± 0.04), the highest values of Haugh unit (61.00 ± 0.07), yolk index (0.37 ± 0.02) were observed in eggs coated with CH/PVA 25:75 wt%. But there was no difference in 50 or 70 wt% PVA significantly. Therefore, the CH/PVA blended coatings containing around 50–75 wt% PVA, as egg biodegradable packaging, can be used to extend the shelf life for 2–3 weeks at ambient temperature.

## Graphical abstract

Aiming to determine the relative ratios of chitosan (CH 4 w/v%) and polyvinyl alcohol (PVA 5 w/v%) in the composition of blended coatings, an experiment was conducted with six treatments (*r* = 3) including different ratios of CH/PVA (0:0; control, 100:0, 75:25, 50:50, 25:75 and 0:100) wt% in a period of 4 weeks of egg storage at ambient temperature, emphasizing eggshell barrier properties. The CH/PVA blended coatings containing around 50–75 wt% PVA, as egg biodegradable packaging, can be used to extend the shelf life for 2–3 weeks at ambient temperature.

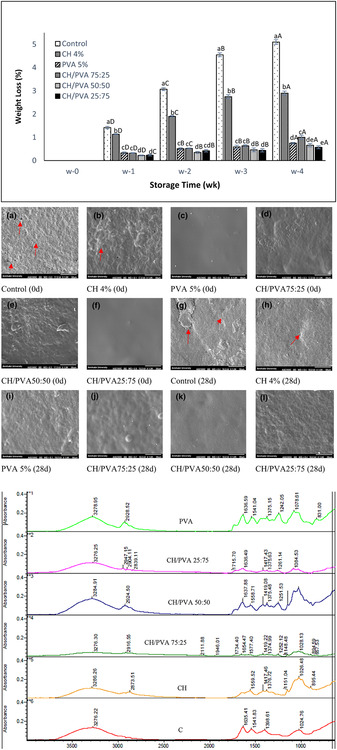

## 1 INTRODUCTION

Eggs as one low‐cost and valuable foodstuffs in the human diet contain a natural source of many nutrients as well as distinguished biological value and are consumed worldwide (Puglisi & Fernandez, 2022). However, eggs are perishable products and decrease in internal quality during handling, due to the higher rate of carbon dioxide escaping through the tiny pores in the eggshells, especially at ambient conditions (Gabriela da Silva Pires et al., 2020). Because of increasing public awareness, there is a growing tendency toward the progressive effective methods to maintain and enhance the egg's internal quality. On the other hand, considering the environmental problem‐related concerns of using oil‐based synthetic polymers, edible coatings as an eco‐friendly technology are nowadays applied to many food products to control gas exchanges, preventing the spread and penetration of microbial contamination as well as oxidative degradation (Dhall, 2013). Almost in all of the previous related studies, the coated eggs represented desirable impacts in egg shelf life extension during storage when compared to uncoated ones (Caner et al., 2022; Caner & Yüceer, 2015; Gabriela da Silva Pires et al., 2020; Pires et al., 2020; Rachtanapun et al., 2022; Yüceer & Caner, 2014).

Chitosan, the second‐most prevalent polysaccharide in the world, which is produced by the alkali deacetylation process of chitin, is a natural, nontoxic, biodegradable, and biocompatible polymer, with antimicrobial properties (Awuchi, Akram, et al., 2022; Awuchi, Morya, et al., 2022; Egbuna et al., 2021). Also, barrier properties and superior film‐forming and coating abilities have been proven for this biopolymer (Vlad‐Bubulac et al., 2022). Chitosan films have permeability to CO_2_ and O_2_ and desirable mechanical properties. However, the fragility of the material and its high permeability to water vapor restrict its application in food packaging (Elsabee & Abdou, 2013). Therefore, various approaches have been examined to improve their physical and functional properties (Vargas et al., 2009; Wang et al., 2020; Wardhono et al., 2022; Yüceer & Caner, 2020). A simple mechanical blending of polymers and producing a composite coating is one effective strategy to overcome the poor mechanical properties of a single material (Bonilla et al., 2014). However, bio‐composites of chitosan with other materials with pioneer or modified characteristics including polyvinyl alcohol (PVA) have been formerly studied (Vlad‐Bubulac et al., 2022; Wardhono et al., 2022).

Polyvinyl alcohol is a nontoxic, easily biodegradable, and thermoplastic synthetic polymer achieved from the hydrolysis of poly (vinyl acetate) and is one of the most usable synthetic polymers mixed with chitosan (Choo et al., 2016; Suganthi et al., 2020). Because of presenting hydroxyl functional groups in PVA molecular chains and abundant amine and hydroxyl groups in chitosan, they can potentially blend by forming hydrogen bonds (Hu et al., 2013; Santos et al., 2014). The functionality purpose of blending chitosan with PVA is to maximize composite coating performance (Wardhono et al., 2022). It was reported that chitosan improves the mechanical properties of PVA by increasing Young's modulus (Olarte‐Paredes et al., 2021). Meanwhile, Wardhono et al. (2022) reported that PVA improved the chitosan films' tensile strength and elongation at break. In addition, their morphological findings showed that no irregularities were detected in CS/PVA blended films, representing high compatibility with both polymers.

The unique characteristics and blending ratios of the polymers, the condition of the mixing process, the type of solvent, and other components in the formulation affect the result of the intended formulation (Wardhono et al., 2022). In this regard, chitosan addition to the blended film containing chitosan (CH): PVA strongly reduced the film stretchability while increasing the film rigidity and resistance to fracture (Bonilla et al., 2014). In a similar study, El‐Hefian et al. (2011) reported that blending of CH: PVA improved tensile strength, in a way improved with increasing PVA content by up to 40%. Also, water absorption in PVA:CH composite films can be controlled by variations in their ratios. As the PVA ratio grows in the blends, the surface tension increases. Blending the PVA with chitosan progresses the tensile strength, flexibility, bulk, and surface hydrophilicity of the composited films (Bahrami et al., 2003).

The aim of this work was to investigate the physical and mechanical properties of edible bio‐composite coatings in different ratios of PVA: CH (0:0; control, 100:0, 75:25, 50:50, 25:75, and 0:100 wt%) on internal quality parameters and eggshell morphology during 28‐day storage at ambient temperature with emphasis on barrier properties. Besides, the obtained results were matched to confirm with scanning electron microscopy (SEM) images and Fourier transform infrared (FTIR) spectroscopy.

## 2 MATERIALS AND METHODS

### 2.1 Materials

Chitosan (CH) white‐colored powder brought from shrimp shells was purchased from the Nano Novin Polymer Co. with a high molecular weight of 310–375 KDa and an 85% degree of deacetylated. Polyvinyl alcohol (PVA), fully hydrolyzed (average Mw: 85–146 KDa) in the form of a white powder was delivered by the Sigma‐Aldrich Co. Glacial acetic acid and glycerol (99%) were procurement from Merck. All chemicals used were of analytical grade; in addition, the solutions were freshly prepared in all of the experiments.

Fresh, feces‐free, without surface cracks, white shell eggs (from 28‐week‐old Hy‐line White hens) with an average weight of 55 ± 5 g were obtained from a commercial layer farm at Mazandaran, Iran.

### 2.2 The preparation of coating solutions

According to the method proposed by Olewnik‐Kruszkowska et al. (2019), in brief, a solution of CH 4% w/v was produced by dissolving chitosan and acetic acid in distilled water (4 g in 100 mL of 1% aqueous acetic acid solution), using a magnetic stirrer for 2 h at 80°C and subsequently overnight at room temperature. Glycerol (30 wt%) was added to the solution as a plasticizer. Also, to prepare the 5% w/v PVA solution, a determined volume of PVA powder was dispersed and then fully dissolved in distilled water at 80°C by using a mechanical stirrer. Next, the CH 4% w/v solution was blended with the PVA 5% w/v solution in proportions of 75:25, 50:50, and 25:75 on the basis of the predetermined composite coating (Table 1).

**TABLE 1:** Composition of the coating material in solutions.

Eggs	Formulations	Proportion (%)	
		CH 4% w/v	PVA 5% w/v
Uncoated	C	‐	‐
Coated	CH	100	‐
	PVA	‐	100
	CH/PVA 75:25	75	25
	CH/PVA 50:50	50	50
	CH/PVA 25:75	25	75

All of the prepared coating solutions were used to coat the eggs with the immersing procedure explained by Rachtanapun et al. (2022). Before coating, eggs were rechecked for possible surface cracks and breakage initially, and then egg weight was recorded. Eggs were wiped with the coating polymer material by being dipped into the coating solutions (separated and mixed solutions) for 1 min. Once dry and ensured complete coating, the treated eggs were stored at ambient temperature for 4 weeks of storage. Uncoated eggs were also stored under the same conditions. Coated and uncoated eggs were weekly examined for internal quality during 28 days of storage.

### 2.3 Measurement of egg internal quality parameters

The height of the albumen and yolk and the width of the yolk were measured with a tripod micrometer (AMES S‐6428) and a digital caliper (Digital INSIZE), respectively. All eggs were weighed at the initial and as well as end of each weekly storage interval, with a digital balance (HL 300, AND). Haugh unit (HU) values were calculated as 100 log (*h* − 1.7*w* ^0.37^ + 7.6), where *h* is the albumen height (mm), and *w* is the weight of the egg (g) (Haugh, 1937). The yolk index (YI) was calculated as the ratio of yolk height (mm) to yolk width (mm) (Ryu et al., 2011). The average weight loss (WL) percentage was calculated as the difference between initial and final egg weights divided by initial egg weights and multiplied by 100 (Xu et al., 2017). The pH of the albumen separated from the yolk was measured using a pH meter (Model AZ 86502). In all measurements, five replicates of coated and uncoated treatments were used at each assessment interval.

### 2.4 Eggshell morphological assessments

A scanning electron microscope (SEM; AIS‐2100, Seron Technology) was used to evaluate the eggshell morphology at the initial and the end of storage. The Fourier transform infrared–attenuated total reflectance (FTIR‐ATR) spectra of the outer surface of eggshells were recorded using an FTIR spectrophotometer (Thermo Fisher Scientific) with attenuated total reflectance (ATR) accessories in the frequency range of 500–4000 cm^−1^.

### 2.5 Statistical analysis

A two‐way analysis of variance (ANOVA) using SPSS software version 20.0 was used to determine differences in the means of data. Statistically significant differences found (*p* < .05) were evaluated using Duncan's multiple range tests. Five replicates for each treatment were used for the evaluations.

## 3 RESULTS AND DISCUSSION

### 3.1 Weight loss (WL)

**FIGURE 1:**
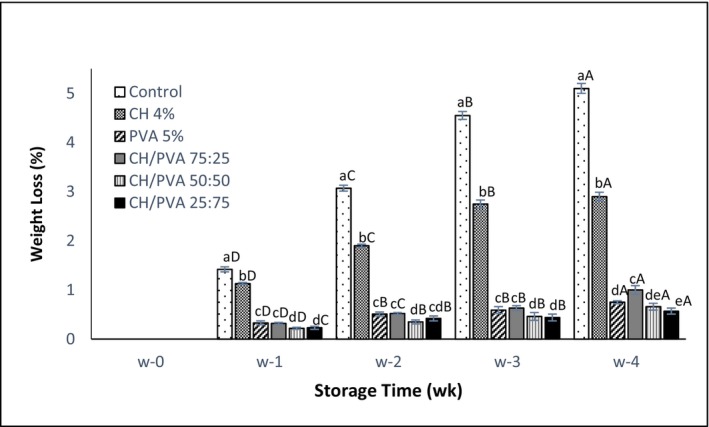
Effects of experimental treatments on weight loss (%) during 4‐week storage. A‐D indicates the significant difference of each treatment during 4‐week storage, a‐d indicates the significant difference of the treatments in each time period storage.

The escape of gases causes many adverse internal physical and chemical changes, which ultimately lead to a deterioration in the quality of the eggs. Therefore, determining the WL values is one of the effective indicators in assessing the interior quality of eggs (Xu et al., 2018). Weight loss (WL) had an upward trend ranging from 0.22% to 5.10% during the retention period for all samples, as shown in Figure 1. While the WL sharply increased throughout storage in uncoated and CH 4% groups, at a similar time, the average values of WL of eggs coated with CH/PVA 50:50 and CH/PVA 25:75 samples were much lower than others till the 4th week (*p* < .05). However, the WL of CH/PVA 25:75‐coated eggs was approximately 16% less than that of CH/PVA 50:50 (0.57% vs. 0.66%) at the end of the storage.

Considering the results exhibited in Figure 1, it was easily found that egg WL was affected by storage time and gradually progressed in treated samples during 28‐day storage. These trend changes were described as the exchange of CO_2_ and water vapors between albumin and the surrounding environment via pores and micro‐cracks on the eggshells (Suresh et al., 2015). This also summarily states that the water vapor permeability of the eggshell was lowered by the filling of eggshell pores with the coating materials (Caner et al., 2022). The effect of the coating material's high resistance to water vapors and the resulting lower evaporation rate caused the coated eggs to show the least weight loss, as described by Rachtanapun et al. (2022).

The barrier property of the coatings, which is related to the weight loss of eggs, is a function of their mechanical properties. In the present research, eggs coated with CH/PVA composite coatings showed less weight loss. Wardhono et al. (2022) reported that according to the results of the mechanical test, PVA improved the blend strength CH/PVA, which could be due to the interaction between the hydroxyl and amine groups of chitosan and hydroxyl groups of PVA. In this study, the lowest WL% was observed in eggs with the highest polyvinyl alcohol ratio in the blended coating (*p* < .05). The CH/PVA blended coating delayed egg weight loss by sealing eggshell pores with the highly water‐resistant coating material, which decreased the evaporation rate of the internal content of the eggs. The prevention of egg weight loss using highly water‐resistant coating materials has also been previously reported by Rachtanapun et al. (2021). In concurrence with our findings, results in research of Bahrami et al. (2003) and El‐Hefian et al. (2011) indicated that blended films have higher tensile strength than pure CH and PVA films. Blending leads to an intermolecular interaction between two polymers and this improves the mechanical strength of the blends. Also, by increasing the PVA content (up to 40%) in the blend, the flexibility of the films was increased.

### 3.2 Haugh unit (HU), yolk index (YI), and albumen pH

Haugh unit and yolk index are two main parameters for evaluating egg quality based on egg proteins (Sheng et al., 2018). The values of these parameters, along with albumen pH, are cumulatively listed in Table 2. According to the United States Standards for Quality of Individual Shell Eggs, the HU values are as follows: AA >72, A from 72 to 60, B from 59 to 31, and C ≤30. In newly laid eggs, HU values are between 75 and 85, while due to the decomposition of organic compounds of proteins and the consequent decrease in albumin height, the HU values gradually decline during storage (Bhale et al., 2003). Several studies have shown that HU decreased as storage duration increases and that the usage of coatings delays this decline (Jirangrat et al., 2010; Rachtanapun et al., 2022). In addition, as a result of the evaporation, the pH of the egg contents rises and leads to the proteolysis of dense proteins (Omana et al., 2011).

**TABLE 2:** Changes of means in Haugh unit (HU), yolk index (YI), and albumen pH during storage.

Parameters and treatments	Storage time (day)				
	0	7	14	21	28
HU					
C	83.74 ± 0.00^aA^	60.64 ± 0.04^dB^	41.07 ± 0.19^eC^	29.16 ± 0.09^eD^	28.28 ± 0.11^eD^
CH 4%	83.74 ± 0.00^aA^	68.60 ± 0.01^cB^	60.86 ± 0.09^dC^	48.14 ± 0.03^dD^	48.13 ± 0.20^dD^
PVA 5%	83.74 ± 0.00^aA^	73.13 ± 0.04^bB^	73.16 ± 0.02^aB^	58.65 ± 0.11^cC^	56.50 ± 0.17^cC^
CH/PVA 75:25	83.74 ± 0.00^aA^	72.93 ± 0.03^bB^	68.58 ± 0.04^cC^	62.65 ± 0.08^aD^	60.55 ± 0.09^abD^
CH/PVA 50:50	83.74 ± 0.00^aA^	75.73 ± 0.04^aB^	69.84 ± 0.01^bcC^	60.38 ± 0.03^bD^	60.30 ± 0.05^abD^
CH/PVA 25:75	83.74 ± 0.00^aA^	74.95 ± 0.04^aB^	71.18 ± 0.05^bC^	63.58 ± 0.10^aD^	61.00 ± 0.07^aD^
YI					
C	0.48 ± 0.00^aA^	0.37 ± 0.01^cB^	0.30 ± 0.01^eC^	0.28 ± 0.03^dCD^	0.27 ± 0.04^eD^
CH 4%	0.48 ± 0.00^aA^	0.39 ± 0.00^abB^	0.35 ± 0.02^dC^	0.33 ± 0.02^cCD^	0.30 ± 0.03^dD^
PVA 5%	0.48 ± 0.00^aA^	0.40 ± 0.00^aB^	0.42 ± 0.01^aB^	0.37 ± 0.03^abC^	0.34 ± 0.03^cD^
CH/PVA 75:25	0.48 ± 0.00^aA^	0.38 ± 0.01^bcB^	0.38 ± 0.02^cB^	0.37 ± 0.01^bBC^	0.35 ± 0.03^bcC^
CH/PVA 50:50	0.48 ± 0.00^aA^	0.41 ± 0.00^aB^	0.39 ± 0.02^bcBC^	0.38 ± 0.02^abCD^	0.36 ± 0.04^abD^
CH/PVA 25:75	0.48 ± 0.00^aA^	0.40 ± 0.01^aB^	0.40 ± 0.01^bBC^	0.39 ± 0.03^aCD^	0.37 ± 0.02^aD^
Albumen pH					
C	8.21 ± 0.01^aD^	8.71 ± 0.02^aC^	8.80 ± 0.03^aB^	8.95 ± 0.02^aA^	8.88 ± 0.04^aAB^
CH 4%	8.20 ± 0.01^aD^	8.54 ± 0.01^bC^	8.66 ± 0.02^bB^	8.70 ± 0.04^bAB^	8.77 ± 0.03^bA^
PVA 5%	8.21 ± 0.01^aD^	8.31 ± 0.02^cB^	8.35 ± 0.02^cB^	8.38 ± 0.04^cAB^	8.41 ± 0.04^cA^
CH/PVA 75:25	8.20 ± 0.01^aC^	8.33 ± 0.03^cB^	8.38 ± 0.04^cAB^	8.40 ± 0.01^cA^	8.44 ± 0.03^cA^
CH/PVA 50:50	8.21 ± 0.01^aC^	8.23 ± 0.02^dB^	8.30 ± 0.01^dAB^	8.30 ± 0.05^dAB^	8.33 ± 0.04^dA^
CH/PVA 25:75	8.21 ± 0.01^aC^	8.23 ± 0.01^dAB^	8.25 ± 0.02^eAB^	8.27 ± 0.03^dAB^	8.30 ± 0.04^dA^

*Note*: Means in the same column with different lowercase letters (a–d), and means in the same row with different capital letters (A–D) are significantly different (*p* < .05).

Abbreviations: C, control; CH, chitosan; PVA, polyvinyl alcohol.

Haugh unit values in uncoated eggs showed a sharp drop and fell from 60.64 (grade A) at the end of the first week to 41.07 (grade B), and 28.28 (grade C) at the end of 14 and 28 days of the storage period, respectively. The disruption of the ovalbumin structure, which is attributed to the increasing albumin pH (Omana et al., 2011), thins albumen and decreases the Haugh unit value during storage (Sheng et al., 2018).

Likewise, the components proportions of the mix coating solution had no effect on the shelf life, so at the end of storage time, all of the CH/PVA composite‐coated eggs had the A grade. From the point of view of the HU grading, the CH/PVA composite coating extends the shelf life up to at least 3 weeks longer at ambient temperature. As shown in Table 2, the undesirable changes in HU values of uncoated eggs as well as to some extent coated with CH 4% and PVA 5% were similar to changes in their WL throughout storage, which can be a reflection of the barrier properties of the composite coatings.

The oxygen permeability (OP) and water vapor permeability (WVP) of coating materials are of considerable importance in food preservation. Low OP exhibits great oxygen barrier abilities (Liu et al., 2017). Liu et al. (2017) investigated the mechanical properties of PVA/CH blended films with various CH contents (0, 20, 25, and 30 wt%). Results showed that when the CH proportion is 30 wt%, it reduces the ability of the blended films to act as oxygen barriers, as was also observed when water vapor permeability was determined. The hydrophilic temper of CH promotes the transport of water molecules via the film, and blended films with higher CH proportions display lower crystallinities. Almost similar results have been obtained in other studies that were mentioned earlier (Bahrami et al., 2003; El‐Hefian et al., 2011). In accordance with these results, in our findings, CH/PVA blended coatings with 25 wt% CH had the best efficiency in terms of barrier capabilities. Among all of the mixed coatings, the barrier abilities reduced simultaneously, as grew the share of CH in the blended coating from 25 wt% to 75 wt%. One of the most interesting results in these studies concerned the interaction between CH and PVA. Olarte‐Paredes et al. (2021) reported that CH improves the mechanical properties of PVA; indeed, it increases its Young's modulus. These results were concordant with our findings, with this explanation that the best mechanical properties were observed in the CH/PVA 25:75 wt%, as reflected in the egg internal quality parameters.

The numerous pores on the outer surface of the eggshell facilitate and accelerate the transfer of water vapor and carbon dioxide, which ultimately affects the internal quality of the egg. During storage, the pH of the egg yolk changes slightly due to the release of carbon dioxide and the transfer of water vapor from the egg albumen to the yolk, which affects the albumin quality (Keener et al., 2000).

Albumen pH is another parameter of chemical changes in eggs during storage. Table 2 exhibits the changes in albumen pH at 4 weeks. Albumen pH increased in uncoated eggs from 8.21 to 8.88. At the same time, the least albumen pH changes were observed in CH/PVA 50:50 and CH/PVA 25:75 blended‐coated eggs (*p* < .05). The pH of albumen enhances because of the hydrolysis of carbonic acid and subsequently, carbon dioxide escapes via the pores of the eggshell (Soares et al., 2021). The CH/PVA blended‐coated samples displayed a lower pH than the uncoated egg, which underlines that the coating materials were extremely effective at preserving egg internal quality within storage time due to an efficient barrier property against the loss of CO_2_ and eggshell pore sealing of the coating material, avoiding changes in the pH of the egg albumen during storage (Caner et al., 2022). As the acidity of albumen changes during storage, the viscosity decreases due to albumen decomposition (Soares et al., 2021), the results of which are also appeared in the changes in HU values.

### 3.3 Outer eggshell morphological assessments

#### 3.3.1 Scanning electron microscope (SEM)

**FIGURE 2:**
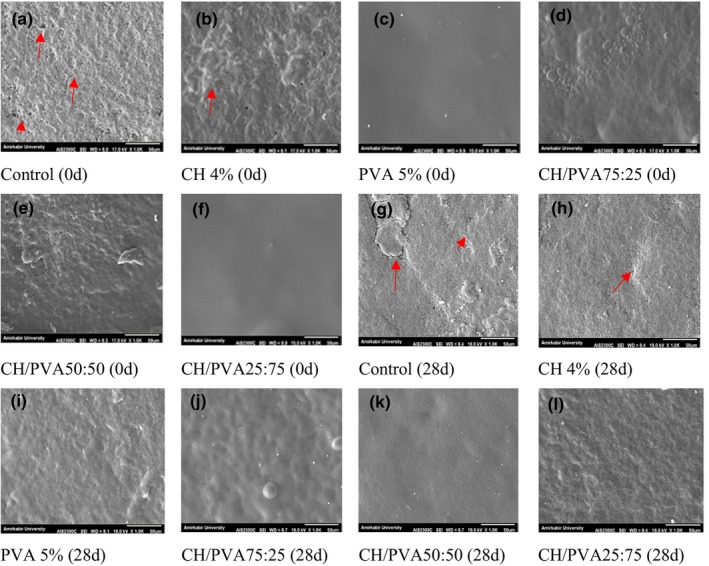
Scanning electron microscopy (SEM) images of eggshell's outer surface at days 0 and 28 (Scale bar = 50 μm). Arrows show some micro‐pits, wrinkles, and discontinuity in images.

The use of biopolymers in the coating of eggs can prevent the exchange of gases between the interior and the surrounding spaces as well as microorganisms' penetration of the egg by filling numerous pores on the eggshell surface, extending eggs' shelf life (Xu et al., 2018). So, it is inferred that the barrier properties of eggshells strongly depend on the sealing quality of the eggshell surface. The microscopic images of the eggshell's outer surfaces are exhibited in Figure 2. Some of the early micro‐cracks probably disappeared on the eggs after immersion of the eggs in coating solutions.

It has been proven that if the outer surface structure of the eggshell is destroyed for any reason, the ability to block the eggshell is surely weakened, resulting in changes in the internal quality indicators (Xu et al., 2018). Scanning electron microscope (SEM) images in Figure 2 exhibit a more uniform structure of coated eggshells than uncoated ones. PVA coating exhibited good structural integrity, smoothness, and crack‐free states. On day 28, as pointed out by arrows, some micro‐pits, wrinkles, and somewhat discontinuity were clearly expanded on the eggshells of uncoated and coated with CH 4%. The blending of CH and PVA coatings solutions at different proportions gave rise to a distinct morphology, in which homogeneous and uniform materials were observed on the surface of the eggshells. SEM images of blended coatings demonstrated a lower porosity surface morphology at CH/PVA as compared to others. One of the most likely reasons for these results may be that PVA/CH material has synergistic properties and there is good adhesion between the PVA/CH materials (Olarte‐Paredes et al., 2021). However, when the CH proportion is increased, it appears slightly as rough areas, due to the CH molecules disrupting the compact structure of the PVA coating (Liu et al., 2017).

#### 3.3.2 Fourier‐transform infrared spectroscopy (FTIR)

**FIGURE 3:**
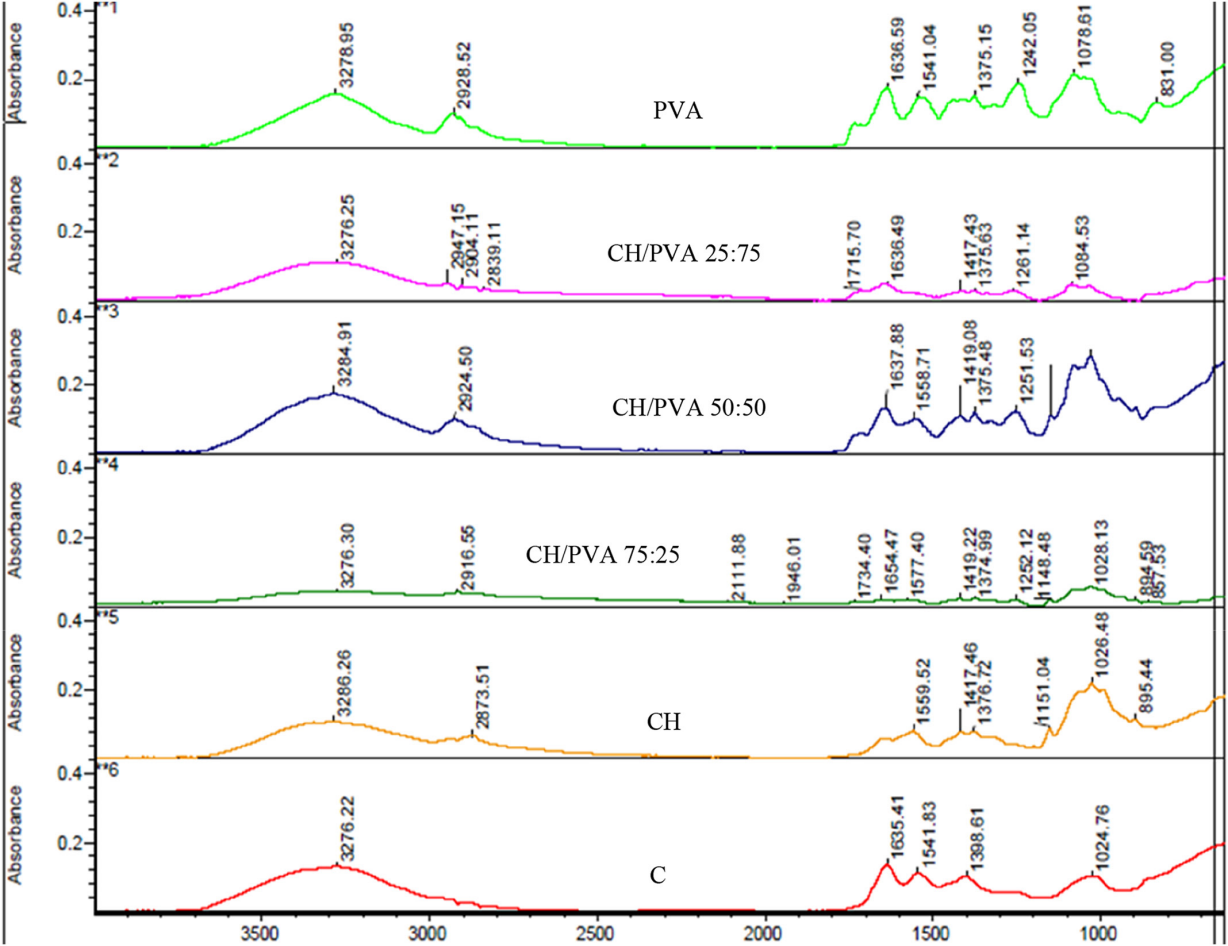
Fourier transform infrared (FTIR) spectra of eggshell surface coatings (CH, PVA, and blends of CH/PVA) at day 28.

Fourier‐transform infrared spectroscopy is an easy, fast, suitable, and cost‐effective method to identify specific molecular structures and recognize the occurrence of different functional groups present in a sample (Wardhono et al., 2022). In the present study, FTIR analysis was made for CH‐ and PVA‐based coating solutions in order to investigate the compatibility and the interactions in the CH/PVA blended coatings. The FTIR spectra of CH and PVA and the blended coatings are exhibited in Figure 3.

In the spectrum corresponding to pure CH, the absorption peak in the range of 1026–1151 cm^−1^ is assigned to the saccharide structure and glycosidic bond, while the band around 1559 cm^−1^ belongs to the bending of the acetamido group in chitosan. Also, the CH strong peak at 3286 cm^−1^ represents the OH absorption bond, as described by Li et al. (2019). From that side, the spectra attributed to the pure PVA coating, the moderate height bands of approximately 3278 and 1636 cm^−1^ belong to the stretching and bending vibrations of the hydroxyl group, respectively (Liu et al., 2017). Other peaks observed at 1242 and 2928 cm^−1^ were assigned to CHOH stretched vibration and the bending vibrations of C‐H bonds, respectively, in concordance with the results previously reported (Li et al., 2019).

A similar band over 3000 cm^−1^ was obtained in CH, PVA, and CH/PVA coatings. This was assigned to ‐OH stretching adsorbed water on the samples, while the peaks at 3000–2800 cm^−1^ can be attributed to C‐H stretching vibration commonly observed in CH and PVA. Several moderate height peaks at around 1559, 1417, 1376, 1151, and 1026 cm^−1^ were also exhibited in the spectrum of CH, signifying the presence of N‐H, C‐H, C‐C, C‐N, and C‐O bonds, respectively. Meanwhile, the peaks at around 1375, 1078, and 831 cm^−1^ seen in the spectrum of PVA were probably related to the C‐H, C‐O, and C‐C bonds, respectively. It is interesting to note that almost all the bands observed in the spectrum of CH and PVA were present in the PVA/CH spectrum indicating that the components of the CH/PVA blended coating were successfully combined (Budlayan et al., 2022). The bands of about 1400 cm^−1^ observed for different weight fractions of CH/PVA (at 1419 cm^−1^ for 75:25, at 1419 cm^−1^ for 50:50, and at 1417 cm^−1^ for 25:75) are attributed to C=N pyridine ring vibrations. This confirmed the interaction between the PVA and CH. The peak intensity increased with enhanced PVA contents, because of hydrogen bonding between the hydroxyl groups of PVA and the hydroxyl, or amine group of CH in the blended coatings (Cao et al., 1998).

## 4 CONCLUSION

The composite of CH/PVA was developed in different blend ratios. FTIR spectroscopy and SEM microscopy confirmed the successful integration of the CH and PVA. Meanwhile, the CH/PVA blends represented better morphological and mechanical properties than pure CH or PVA. They delay perishable internal chemical changes due to good barrier abilities, as exhibited by reductions in weight loss as well as prevention of sharp decline in the HU and YI indicators. The CH/PVA blended coatings demonstrated an advantageous impact on the preservation of coated eggs' quality and freshness throughout the storage period, in which bio‐composite coatings were very efficient in covering the pores or micro‐cracks that existed in the shell, helping enhance the internal quality. Indeed, they extended egg shelf life by at least 2–3 weeks compared to uncoated ones. One of the most interesting results concerned the interaction between CH and PVA. Increasing the PVA ratio in the blends improved mechanical properties but the best mechanical properties were observed in the CH/PVA 25:75 wt% sample, a CH/PVA coating material blend with a 75% weight ratio of PVA in comparison with control and other coated groups. So, it may be one of the best choices for preserving the quality and extending the shelf life of table eggs for up to 28 days in ambient conditions, with acceptable scores of eggshell morphological analysis and internal chemical qualities.

## AUTHOR CONTRIBUTIONS

**Dariush Khademi Shurmasti:** Conceptualization (equal); data curation (equal); methodology (equal); project administration (equal); software (equal); validation (equal); visualization (equal); writing – original draft (equal). **Pezhman Riazi Kermani:** Conceptualization (equal); data curation (equal); formal analysis (equal); investigation (equal); methodology (equal); project administration (equal); resources (equal); software (equal); supervision (equal); validation (equal); visualization (equal); writing – original draft (equal); writing – review and editing (equal). **Maryam Sarvarian:** Conceptualization (equal); data curation (equal); formal analysis (equal); investigation (equal); methodology (equal); project administration (equal); writing – review and editing (equal). **Chinaza Godswill Awuchi:** Data curation (equal); formal analysis (equal); methodology (equal); project administration (equal); validation (equal); visualization (equal); writing – review and editing (equal).

## CONFLICT OF INTEREST STATEMENT

The authors declare that they have no conflict of interest.

## ETHICS STATEMENT

The study does not involve any human or animal testing.

## Data Availability

Data used for this review are available on request through the corresponding author, although all the relevant data have been provided here.
